# Trophoblast-Specific Expression of Hif-1α Results in Preeclampsia-Like Symptoms and Fetal Growth Restriction

**DOI:** 10.1038/s41598-019-39426-5

**Published:** 2019-02-26

**Authors:** Renee E. Albers, Melissa R. Kaufman, Bryony V. Natale, Chanel Keoni, Kashmira Kulkarni-Datar, Sarah Min, Clintoria R. Williams, David R. C. Natale, Thomas L. Brown

**Affiliations:** 1 Department of Neuroscience, Cell Biology and Physiology, Wright State University Boonshoft School of Medicine, Dayton, Ohio 45435 USA; 2 Department of Reproductive Medicine, University of California-San Diego, San Diego, California 92093 USA

## Abstract

The placenta is an essential organ that is formed during pregnancy and its proper development is critical for embryonic survival. While several animal models have been shown to exhibit some of the pathological effects present in human preeclampsia, these models often do not represent the physiological aspects that have been identified. Hypoxia-inducible factor 1 alpha (Hif-1α) is a necessary component of the cellular oxygen-sensing machinery and has been implicated as a major regulator of trophoblast differentiation. Elevated levels of Hif-1α in the human placenta have been linked to the development of pregnancy-associated disorders, such as preeclampsia and fetal growth restriction. As oxygen regulation is a critical determinant for placentogenesis, we determined the effects of constitutively active Hif-1α, specifically in trophoblasts, on mouse placental development *in vivo*. Our research indicates that prolonged expression of trophoblast-specific Hif-1α leads to a significant decrease in fetal birth weight. In addition, we noted significant physiological alterations in placental differentiation that included reduced branching morphogenesis, alterations in maternal and fetal blood spaces, and failure to remodel the maternal spiral arteries. These placental alterations resulted in subsequent maternal hypertension with parturitional resolution and maternal kidney glomeruloendotheliosis with accompanying proteinuria, classic hallmarks of preeclampsia. Our findings identify Hif-1α as a critical molecular mediator of placental development and indicate that prolonged expression of Hif-1α, explicitly in placental trophoblasts causes maternal pathology and establishes a mouse model that significantly recapitulates the physiological and pathophysiological characteristics of preeclampsia with fetal growth restriction.

## Introduction

The placenta is a transient organ that is unique to pregnancy and is composed of trophoblast cells that facilitate the transport of nutrients, gases, and wastes between the mother and fetus^1–5^. If oxygen levels are not regulated properly during gestation, abnormal trophoblast differentiation and development can occur and result in pregnancy-associated disorders^6–10^.

Hif-1 is a critical regulator of oxygen homeostasis in all multicellular eukaryotes and is essential for placental development^11–18^. Hif-1 is a transcriptional activator composed of Hif-1α and Hif-1β (ARNT) subunits, and under very low oxygen (hypoxic) conditions, it regulates genes that control cell growth, differentiation, and metabolism^9,19,20^. Under normoxic conditions, however, the Hif-1α protein is rapidly degraded, thereby rendering it inactive^7,21,22^. A constitutively active form of Hif-1α has been shown to inhibit the differentiation of trophoblast giant cells in cell culture^19^. In addition, elevated levels of placental HIF-1α, beyond the first trimester, have been associated with preeclampsia in humans^10,13,14,23–28^.

Preeclampsia is a devastating disorder that develops in 5–8% of all pregnancies and is a leading cause of maternal and fetal death^14,29,30^. Fetal growth restriction (FGR) is the second leading cause of perinatal mortality and is present in conjunction with preeclampsia in many cases^31,32^. Studies into the biology and development of preeclampsia have focused predominantly on the maternal pathophysiology of pregnancy-induced hypertension; however, such analysis has often overlooked the critical environment in which preeclampsia originates, that of placental hypoxic duress^33^.

## Results and Discussion

**Figure 1:**
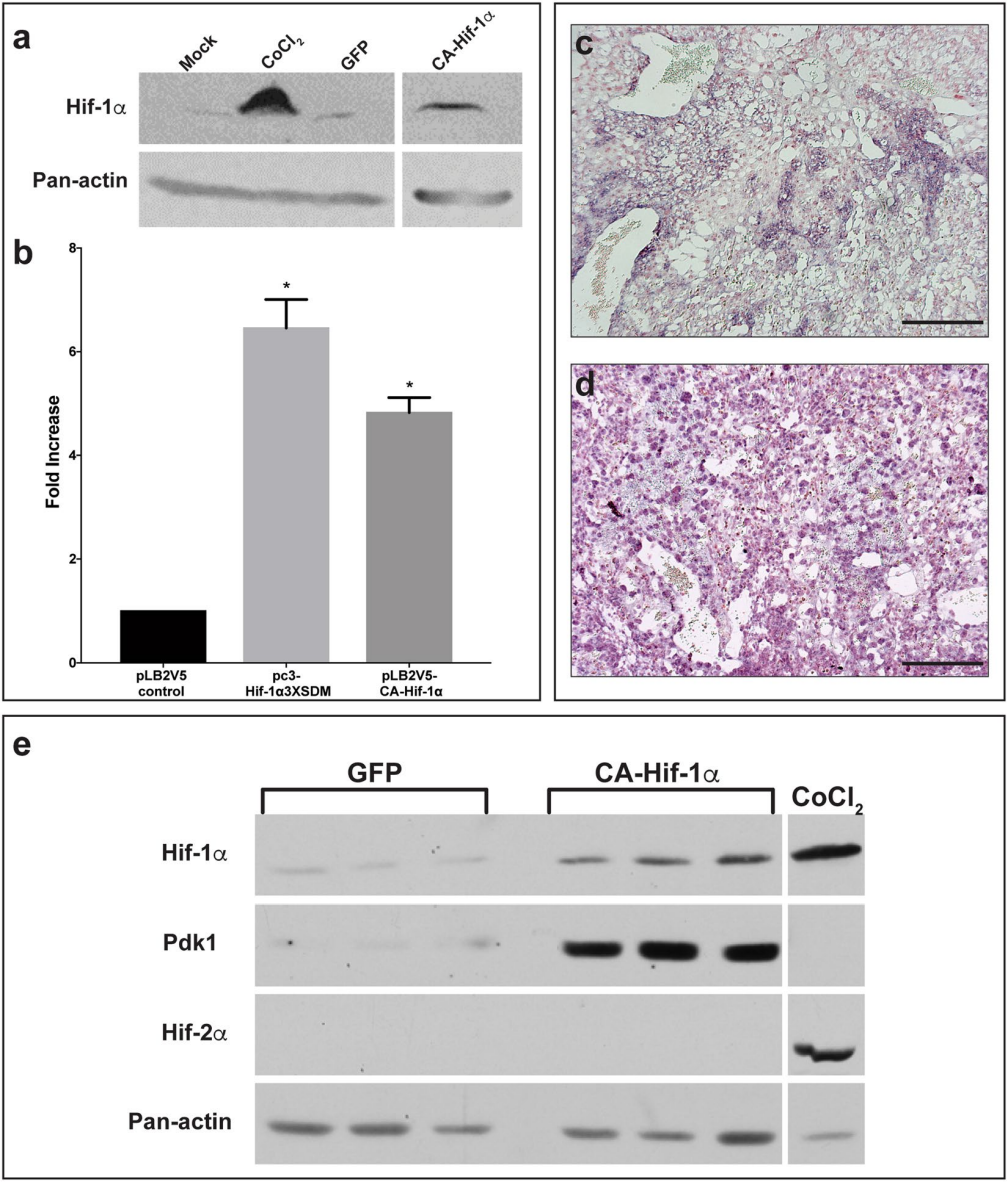
Lentiviral CA-Hif-1α is expressed and activates downstream targets in the mouse placenta following blastocyst infection. (**a**) Hif-1α protein is stably expressed in COS-7 cells under normoxic conditions following treatment with CoCl_2_ or transduction with CA-Hif-1α expression construct, compared to non-transduced (Mock) or GFP-transduced controls (raw data, Fig. S2). (**b**) In luciferase assays using the Pgk1-luciferase reporter, both pc3-Hif-1α3XSDM and the lentiviral pLB2V5-CA-Hif-1α overexpression constructs promoted increased luciferase expression compared to the pLB2V5 control, p < 0.0001 by ANOVA with Tukey’s multiple comparisons test (*). (**c**,**d**) Expression of Hif-1α mRNA was detectable by *in situ* hybridization in E14.5 placentas derived from blastocysts infected with lentiviral particles containing GFP (**c**) or CA-Hif-1α (**d**) expression constructs. Expression was more intense and broadly distributed in CA-Hif-1α placentas. (**e**) CA-Hif-1α placentas collected at E19.5 showed increased expression of Hif-1α protein and its downstream target, Pdk1 compared to GFP controls, but an absence of Hif-2α protein, as detected by Western blot. CoCl_2_-treated COS7 cells were used as a positive control for Hif-1α and Hif-2α protein expression. Pan-actin was used as a loading control (**a**,**e**), (raw data, Figs S3–6) Scale bar = 100 μm.

**Figure 2:**
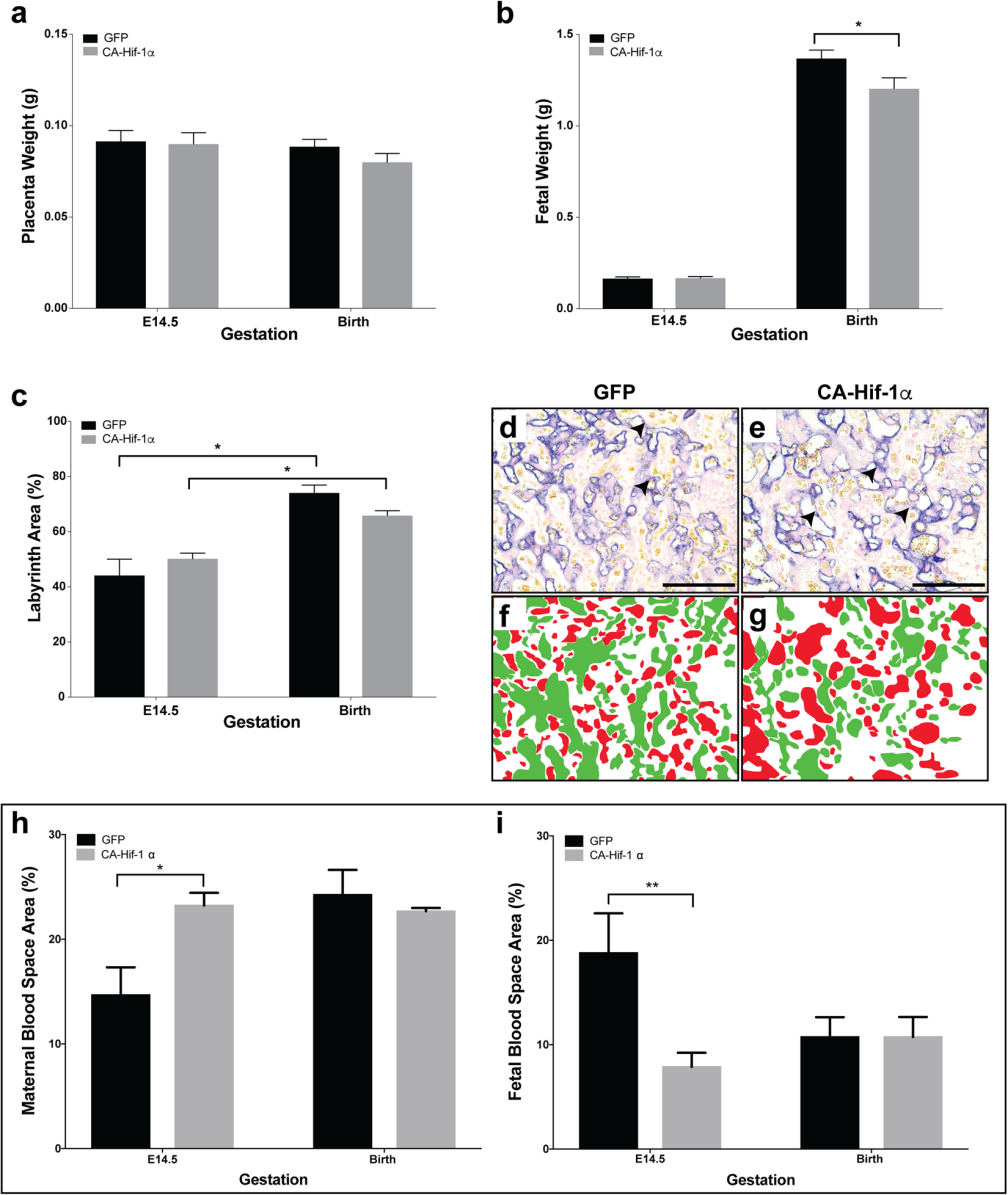
Expression of CA-Hif-1α in trophoblast cells results in reduced fetal weight and altered placental development. (**a**) Placental weights at E14.5 or birth were not significantly altered by CA-Hif-1α expression, compared to GFP controls. (**b**) At E14.5, fetal weights from blastocysts infected with CA-Hif-1α were not significantly different, compared to GFP-infected controls (E14.5 GFP pups: n = 7 pups from 3 pregnancies; E14.5 CA-Hif-1α pups: n = 8 from 4 pregnancies). At birth, however, CA-Hif-1α pup weights (n = 21 pups from 6 pregnancies, avg. 1.20 g) were significantly reduced by 12.10%, compared to GFP control pup weights (n = 23 pups from 8 pregnancies, avg. 1.37 g), (*p = 0.03; by mixed model analysis). (**c**) Assessment of the different layers of the placenta showed that at E14.5 and at birth, the labyrinth layers of CA-Hif-1α and GFP placentas were comparable to each other and that both underwent a statistically significant increase in size between E14.5 and birth (*p = 0.0002; two way ANOVA with Sidak’s Multiple Comparison test). (**d**,**e**) Alkaline phosphatase staining in GFP and CA-Hif-1α placentas at E14.5, respectively, is expressed in sinusoidal TGCs and identifies maternal blood spaces in the labyrinth layer (arrowheads). Pseudo-colouring of fetal (green) and maternal (red) blood spaces in GFP (**f**) and CA-Hif-1α (**g**) images, which when quantified, identified changes in the makeup of maternal and fetal blood spaces in the labyrinth layer, suggesting reduced branching and limited development of the fetal capillary network. This difference was evident at E14.5 but not at birth (**h**,**i**); *p = 0.0255 maternal, **p = 0.0246 fetal by two way ANOVA with Sidak’s Multiple Comparison test. Scale bar = 50 μm.

To address the issue of placental hypoxic duress *in vivo*, we prolonged the expression of Hif-1α, specifically in trophoblasts, using an oxygen-insensitive form of Hif-1α (CA-Hif-1α)^19,34^. To direct trophoblast-specific transgenic expression in mice, we used lentiviral blastocyst transduction and non-surgical embryo transfer^35–40^. This lentivirally-driven gene delivery results in expression of the target gene exclusively in trophoblast cells of the placenta, as indicated by V5 immunohistochemistry, without altering maternal or embryonic tissue (Fig. S1)^38^. To investigate the effects of prolonged CA-Hif-1α, we confirmed the stability of Hif-1α protein in normoxia by Western blot (Figs 1a and S2), while functional activation of a direct and Hif-1α-specific target was confirmed using Pgk1-luciferase (Fig. 1b)^41^. We further validated the prolonged expression and activation of CA-Hif-1α *in vivo* by *in situ* hybridization at embryonic day 14.5 (E14.5) (Fig. 1c,d) and in placental homogenates collected at E19.5 by Western blot (Figs 1e and S3–S6). The absence of detectable Hif-2α protein, an alternative Hif isoform, in E19.5 GFP and CA-Hif-1α placentas indicates that Hif-2α has no redundant, competing or compensatory role (Fig. 1e). In addition, increased levels of Pdk1 (a direct and specific target of Hif-1α) in homogenates of CA-Hif-1α placentas at E19.5, indicates that CA-Hif-1α is functionally active *in vivo* (Fig. 1e)^42^. Our results demonstrate that CA-Hif-1α is stable and transcriptionally active in normoxia in cell culture and is expressed and functionally active in placental trophoblasts throughout gestation in CA-Hif-1α placentas *in vivo*. When we examined gestational outcomes in GFP and CA-Hif-1α mice at E14.5, we found no significant differences in placental or fetal weights (Fig. 2a,b). While the placental weights of GFP and CA-Hif-1α mice at birth were similar; CA-Hif-1α offspring at birth had a statistically significant, 12.10% reduction in weight, compared to GFP controls (Fig. 2b). No differences were noted in litter size (Fig. S7a) or fetal/placental weight ratio (Fig. S7b) between GFP control and CA-HIF-1α mice. Our findings thus indicate that prolonged expression of trophoblast-specific CA-Hif-1α results in fetal growth restriction. It is widely accepted that offspring that were fetally growth restricted are at significantly increased risk of several diseases later in life, in particular, cardiovascular and metabolic issues that may be a result of fetal programming *in utero*^43^. Furthermore, fetal programming is often sexually dimorphic and thus future studies investigating these areas are warranted.

To further evaluate FGR in CA-Hif-1α mice, we investigated placental pathology. The primary role of the murine labyrinth is to facilitate placental and fetal growth via nutrient transport and gas and waste exchange between the maternal/fetal blood spaces^44^. Therefore, it was critical to evaluate the effect of prolonged CA-Hif-1α expression on the relative area of each of the placental layers, as well as maternal and fetal blood spaces. At both E14.5 and birth, there was no statistical difference in the labyrinth and decidual layer areas, relative to total placenta area, when comparing CA-Hif-1α to GFP controls (Fig. S8). When examined individually, however, both GFP and CA-Hif-1α placentas had a statistically significant increase in labyrinth layer size as gestation progressed, as expected. The increase in the labyrinth of GFP control placentas was ~67%, relative to its size at E14.5, however, the increase in labyrinth size in the CA-Hif-1α placentas was only ~31% (Fig. 2c).

Sinusoidal trophoblast giant cells that line the maternal blood spaces express endogenous alkaline phosphatase (AP), thus allowing for their identification (Fig. 2d,e)^45^. The total blood space area (maternal and fetal combined), showed no statistical difference, regardless of gestational age or condition (Fig. 2f–i). The significant differences we observed were in the ratio between maternal and fetal blood spaces (Fig. 2h,i). Maternal blood space area in the CA-Hif-1α placentas was 58% larger at E14.5, though equal at birth, when compared to GFP placentas (Fig. 2h), suggesting that at E14.5, branching of the fetal capillaries was compromised. Supporting this, the fetal blood spaces were smaller and fewer in E14.5 placentas with prolonged CA-Hif-1α (Fig. 2i). Taken together, our analysis indicated that at E14.5, normal branching morphogenesis of the labyrinth in CA-Hif-1α placentas had not taken place and resulted in altered labyrinth morphology with respect to maternal and fetal blood spaces. The decreased fetal blood spaces in CA-Hif-1α placentas at midgestation, but not term, suggest that FGR may be an early manifestation of decreased nutrient transport and/or altered metabolic regulation and that the placenta may have plasticity to maintain fetal survival when under duress.

**Figure 3:**
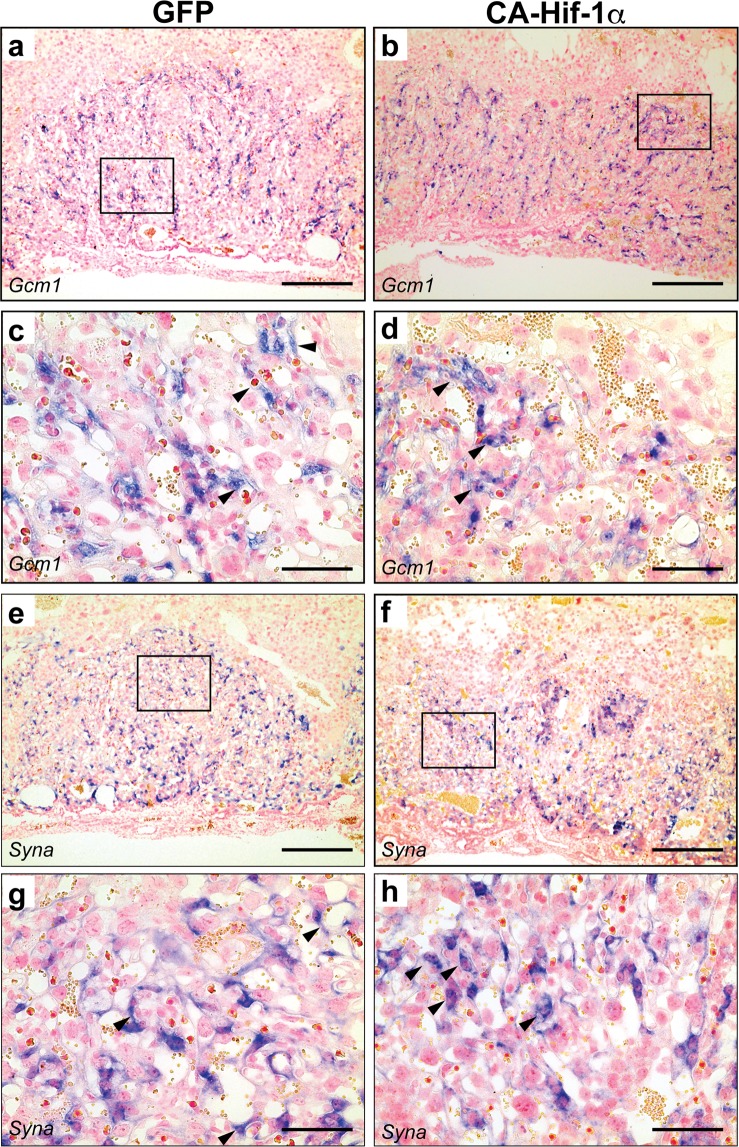
Expression of markers of syncytiotrophoblast demonstrated disorganized labyrinth development in CA-Hif-1α placentas. *Gcm1*, a marker of syncytiotrophoblast layer 2, was detectable in both GFP (**a**,**c**) and CA-Hif-1α (**b**,**d**) E14.5 placentas. In GFP controls, *Gcm1* was associated with fetal blood spaces (**c**, arrowheads) and evenly distributed throughout the labyrinth layer. In CA-Hif-1α placentas, however, *Gcm1* expression was observed in clusters of cells with limited association with blood spaces (**d**, arrowheads). *Syncytin a (Syna)*, a marker of syncytiotrophoblast layer 1, was detectable at E14.5 in both GFP (**e**,**g**) and CA-Hif-1α (**f**,**h**) placentas; however, in CA-Hif-1α placentas, *Syna* expression was less evenly distributed throughout the labyrinth and was also observed in clusters of cells that were not associated with blood spaces (**h**, arrowheads), compared to the GFP controls (**g**, arrowheads). Boxed regions in a,b and e,f are shown at high magnification in c,d and g,h, respectively. Scale bar = 500 μm in (**a**,**b**,**e**,**f**) and = 100 μm in (**c**,**d**,**g**,**h**).

During early placental development, different trophoblast lineages form in two distinct regions, the junctional zone and the labyrinth. Within these regions, distinct trophoblast cell subtypes are present and can be identified morphologically, as well as by *in situ* hybridization (Fig. S9a,b)^45–47^. To determine the effects of prolonged expression of CA-Hif-1α on trophoblast development during pregnancy, we used established trophoblast lineage markers^45–49^. An assessment of labyrinth-specific cell types was conducted by examining the expression of *Epcam*, for labyrinth trophoblast progenitors, as well as *Gcm-1* and *Syna*, for syncytiotrophoblasts (Figs 3 and S10a–d)^50,51^. A labyrinth phenotype was evident in CA-Hif-1α placentas. Relative expression of *Epcam*, in individual cells at the fetal chorionic-mesodermal interface of the labyrinth layer, remained unchanged (Fig. S10a–d). However, *Epcam* staining, that highlighted fetal branching points in the chorionic plate, indicated fewer branch points in E14.5 CA-Hif-1α placentas (Fig. S10a,b), which was more pronounced at birth (Fig. S10c,d). This was also observed and confirmed by isolectin staining, which identifies fetal blood vessels in the labyrinth (Fig. S10e–h)^45^. Syncytiotrophoblast markers *Gcm1* and *Syna* allowed us to investigate the formation of the placental labyrinth (Fig. 3a–h). *Gcm1* expression is typically associated with fetal blood spaces, as seen in GFP control placentas (Fig. 3a,c). In CA-Hif-1α placentas; however, *Gcm1* expression was disorganized, appeared punctate, and was not specifically associated with the fetal blood spaces (Fig. 3b,d). Typically, there is a clear demarcation of the labyrinth layer that is defined by the evenly distributed expression of *Syna*, as observed in the GFP controls (Fig. 3e,g); but in placentas with prolonged CA-Hif-1α, there were less evenly-distributed pockets of *Syna-*positive trophoblast clusters, further indicating a less organized labyrinth (Fig. 3f,h).

**Figure 4:**
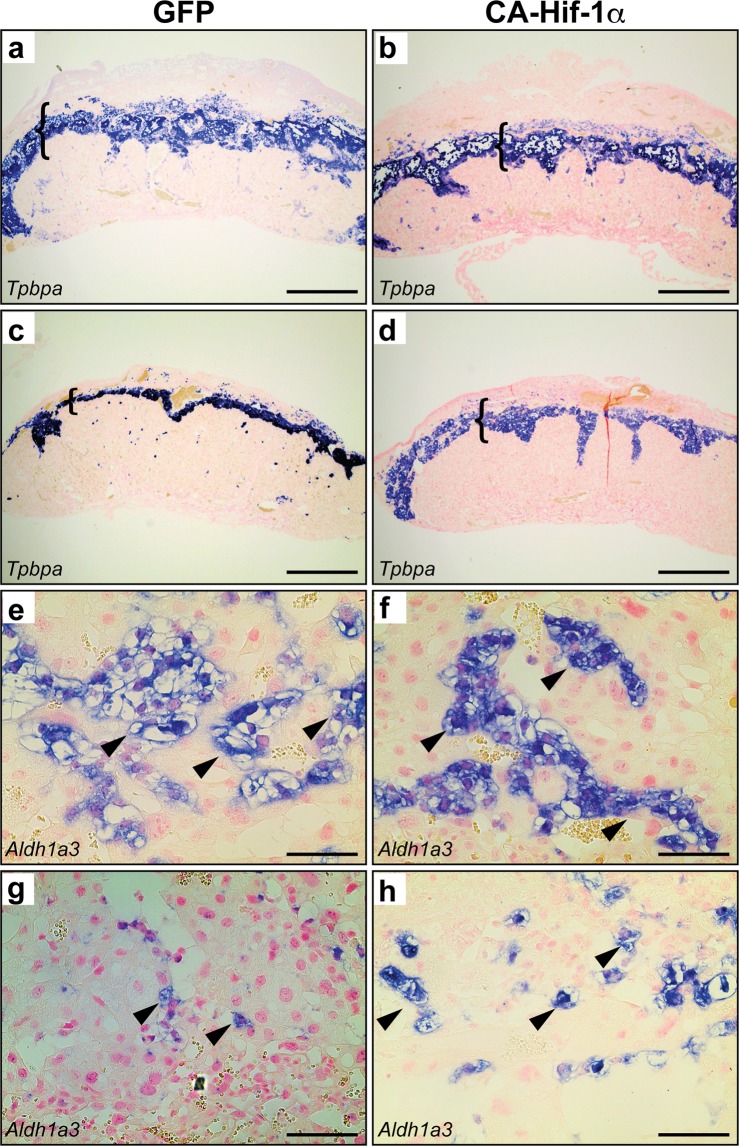
Junctional zone morphology is altered in CA-Hif-1α placentas at E14.5 and birth. *Tpbpa*, a marker of spongiotrophoblast and glycogen trophoblast was detectable in both GFP (**a**,**c**) and CA-Hif-1α (**b**,**d**) placentas and identified the junctional zone at both E14.5 (**a**,**b**) and birth (**c**,**d**). At E14.5, the junctional zone in CA-Hif-1α placentas appeared smaller and more compact than in GFP controls (brackets). While the GFP controls undergo a typical decrease in junctional zone size at birth (**c**), the CA-Hif-1α placentas did not exhibit the same rate of JZ reduction (**d**). *Aldh1a3* identified glycogen trophoblasts (arrowheads) in the junctional zone of GFP (**e**,**g**) and CA-Hif-1α (**f**,**h**) placentas at E14.5 (**e**,**f**) and birth (**g**,**h**). At high magnification, it was evident that at E14.5 in CA-Hif-1α placentas (**f**), glycogen trophoblasts appeared smaller in size compared to GFP controls (**e**), with an increased density of nuclei. By birth, glycogen trophoblasts were mostly absent from the junctional zone of GFP control placentas (**g**); however, they were still present in CA-Hif-1α placentas (**h**). Scale bar = 500 μm in (**a**,**b**,**c**,**d**) and = 100 μm in (**e**,**f**,**g**,**h**).

The junctional zone (JZ) was also evaluated by measuring the relative area of *Tpbpa* expression, which is present in spongiotrophoblasts and glycogen trophoblasts. Identification of the JZ, by *Tpbpa* expression, allowed for the measurement of the areas of the decidua, JZ and labyrinth (Fig. S8). At E14.5, there was a reduction in the relative area of the JZ in CA-Hif-1α placentas, compared to GFP controls (Figs 4a,b and S8). As gestation proceeds, the relative area of the JZ decreases as the labyrinth expands in size. Control GFP placentas at birth displayed the expected reduction of *Tpbpa* expression in the JZ area (Fig. 4c). In contrast, analysis of CA-Hif-1α placentas at birth indicated that they did not undergo the normal decrease in the relative area of *Tpbpa* expression and thus maintained an area similar to that of CA-Hif-1α placentas at E14.5 (Figs 4d and S9). To further assess trophoblast differentiation in the junctional zone, we examined the expression of *Ascl2* (*Mash2*) to identify JZ progenitors^51^. No significant differences were noted in JZ *Ascl2* progenitors at E14.5 (Fig. S11a,b). In addition, *in situ* hybridization of *Aldh1a3* and PAS staining were used to investigate glycogen trophoblasts (Figs 4e–h and S11c–f), while *Prl3b1 (PL2)* was used to demarcate spongiotrophoblasts (Fig. S12a–d)^51–53^. Analysis of glycogen trophoblast cells at E14.5 indicated that, in individual pockets of glycogen cells, the density of nuclei were increased in CA-Hif-1α placentas, compared to GFP controls (Figs 4e,f, S11c–f and S12a–d), which suggested a decrease in cell size. This reduction in glycogen cell size may account for the smaller size of the JZ at E14.5 in CA-Hif-1α placentas. While the populations of glycogen trophoblast cells themselves were not noticeably different at birth, the spongiotrophoblasts were more compact in the GFP controls, indicative of normal placental development; however, in CA-Hif-1α placentas, spongiotrophoblasts still maintained a larger, spongy morphology. Taken together, these characteristics likely contributed to the overall junctional zone phenotype and the difference in JZ size between GFP control and CA-Hif-1α placentas at birth (Figs 4c,d,g,h and S12).

In a normal pregnant mouse, invasive trophoblast giant cells reach the maternal spiral arteries around E10.5 and become exposed to increased levels of oxygen (~12%), leading to the rapid and continuous degradation of Hif-1α protein and resultant loss of Hif-1α activity throughout the rest of gestation. Hif-1α inactivation at this developmental window is spatially and temporally coordinated and is required for trophoblast differentiation, giant cell invasion, and remodeling of the maternal spiral arteries; critical steps needed to facilitate increased blood flow and oxygen to the developing placenta and fetus^45–47,54^. Poorly regulated and reduced levels of oxygen at this invasive developmental time point; however, may prolong the expression of trophoblast Hif-1α and thereby alter critical steps involved in placental development; potentially leading to pregnancy-associated disorders, such as preeclampsia and/or fetal growth restriction.

**Figure 5:**
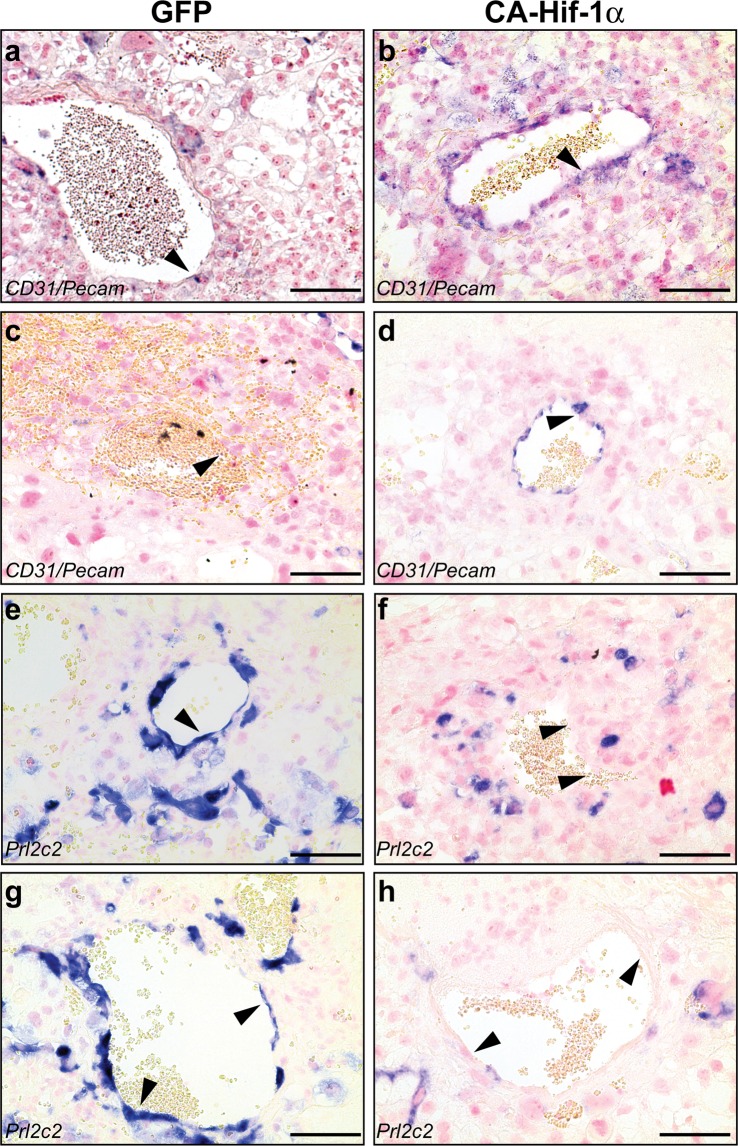
Maternal spiral arteries are not remodelled in CA-Hif-1α placentas. *CD31/Pecam*, a marker of endothelial cells, was nearly undetectable in cells lining the maternal spiral arteries (arrowhead) in GFP E14.5 placentas, as expected (**a**); however, *CD31/Pecam* was clearly present (arrowhead*)* in CA-Hif-1α placentas at E14.5 (**b**). At birth, endothelial cells were undetectable in these vessels in GFP placentas (arrowhead) indicating normal trophoblast remodelling had occured (**c**). However, endothelial cells, which should have been remodelled and replaced by invading trophoblasts, were still *CD31/Pecam* positive and present at birth in the CA-Hif-1α placentas (arrowhead) (**d**). *Prl2c2* is expressed in spiral-artery associated trophoblast giant cells that invade and identify maternal vessels that are undergoing or have undergone remodelling by invading trophoblast cells. At E14.5 (**e**) and birth (**g**), *Prl2c2* expression was evident in GFP controls in cells lining maternal spiral arteries that have undergone normal trophoblast remodeling. In CA-Hif-1α placentas, while some *Prl2c2* positive cells were present, they did not line maternal vessels in the decidua at either E14.5 (**f**) and birth (**h**). The staining pattern of *CD31/Pecam*, combined that of *Prl2c2*, in CA-Hif-1α placentas indicated a lack of appropriate trophoblast remodelling of the maternal spiral arteries. Scale bar = 100 μm.

To determine whether prolonged expression of Hif-1α affected trophoblast giant cell differentiation, a placental assessment was conducted to evaluate trophoblast cells that invade the maternal decidua and the extent of spiral artery remodeling. Healthy spiral artery remodeling includes the breakdown of maternal smooth muscle that lines the artery, as well as a reduction in maternal endothelial cells, which are replaced by invasive spiral artery-trophoblast giant cells (SA-TGCs)^47^. When we prolonged the expression of CA-Hif-1α in trophoblast cells beyond its’ normal developmental window, we observed a continued presence of *CD31 (Pecam1*)-positive maternal endothelial cells (Fig. 5a–d), as well as a dramatic reduction in *Prl2c2* (*Plf*)-positive invasive SA-TGCs (Figs 5e–h and S13a–d) associated with the maternal spiral arteries, both at E14.5 and at birth, compared to GFP controls. Additionally, fewer *Prl2c2*-positive trophoblast cells were associated with the maternal blood canals that deliver blood to the labyrinth in CA-Hif-1α placentas at E14.5 and at birth (Fig. S13a–d). These findings indicated that prolonged expression of trophoblast-specific CA-Hif-1α resulted in the lack of maternal spiral artery remodeling and also suggested that differentiation and/or invasion of *Prl2c2*-expressing TGCs was substantially decreased. Our results suggest that initial alterations in placental development are due to the lack of spiral artery remodeling in response to an inhibition of trophoblast differentiation in the presence of prolonged CA-Hif-1α. This is supported by our previous studies that show that Hif-1α inhibits trophoblast differentiation in rat trophoblast cells^19^. The lack of spiral artery remodeling would likely generate a subsequent hypoxic environment in the junctional zone and labyrinth and the end result would be the development of preeclampsia-like symptoms.

**Figure 6:**
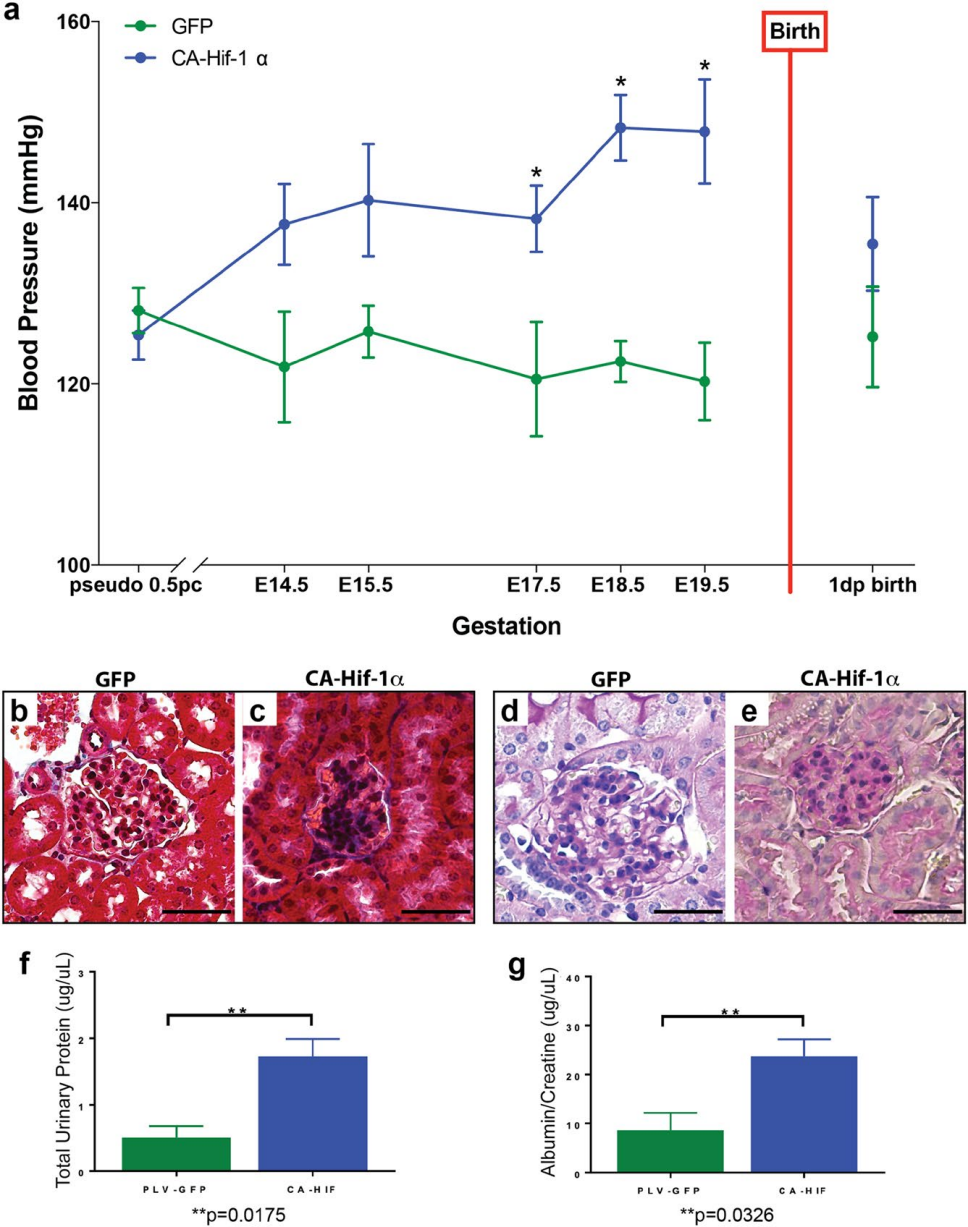
Dams carrying CA-Hif-1α placentas displayed elevated blood pressure and glomeruloendotheliosis. (**a**) Blood pressure in dams was read sequentially throughout gestation at the time points indicated. Dams pregnant with CA-Hif-1α placentas (n = 5–8) displayed elevated blood pressure beginning at E14.5, compared to dams carrying GFP placentas (n = 5–8). At E17.5-E19.5, the difference in blood pressure elevation was statistically significant in CA-Hif-1α mice by Sidak’s multiple comparison test: E17.5, *p = 0.0270; E18.5, *p = 0.0005; E19.5, *p = 0.0006. Following birth, blood pressure of dams pregnant with CA-Hif-1α placentas resolved to levels comparable with dams pregnant with GFP placentas. Kidneys were dissected from pregnant dams carrying GFP or CA-Hif-1α placentas at E19.5 and sections were stained with Masson’s Trichrome (**b**,**c**) or periodic acid Schiff (PAS) (**d**,**e**). Kidneys from dams with GFP placentas appeared histologically normal at E19.5 (**b**,**d**). Maternal kidneys from CA-Hif-1α mice at E19.5, however, showed glomerular damage and had discernable collagen deposition in the glomeruli, as well as occlusion of capillary lumens, as indicated by Masson-Trichrome staining (**b**,**c**). PAS staining further confirmed these results and also indicated that CA-Hif-1α mothers have thinner glomerular basement membranes (**d**,**e**), indicative of glomeruloendotheliosis. Scale bar = 100 μm. To assess kidney damage and function, spot urine samples from E18.5 and E19.5 control GFP (n = 3) and CA-Hif-1α (n = 4) dams were collected and analyzed. (**f**) To determine total urinary protein, the bicinchoninic acid (BCA) protein assay was used (Pierce). (**g**) To determine kidney dysfunction and the detect the presence of proteinuria, albumin and creatinine were measured by ELISA (EXOCELL). Maternal kidneys from GFP controls at E19.5 displayed normal architecture (**b**,**d**). In CA-Hif-1α mice; however, total urinary proteins were significantly increased in CA-Hif-1α mice (**f**). Additionally, the urinary albumin/creatinine ratio (ACR), indicative of proteinuria, was also are significantly elevated in CA-Hif-1α mice compared to GFP control mice (**g**). Collectively, our data demonstrate that glomerular damage and dysfunction occurs in CA-Hif-1α mice.

Based on the placental disorganization and reduced maternal spiral artery remodeling, we hypothesized that maternal blood pressure would be elevated, consistent with the pathophysiology that occurs in human preeclampsia. To examine the effects of trophoblast-specific CA-Hif-1α expression on maternal hypertension, blood pressure was analyzed via VPR tail cuff (Coda, Kent Scientific) in GFP control and CA-Hif-1α mothers throughout and following pregnancy. Although telemetry may be a preferred form of blood pressure measurement, analysis by non-invasive VPR tail cuff is also an accepted alternative and has been shown to produce comparable and similar blood pressure measurements in non-simultaneous recordings^55,56^, as this study was. Our analysis of blood pressure data indicate that CA-Hif-1α pregnant mice developed significantly elevated maternal hypertension, when compared to GFP controls, beginning at E17.5, that remained elevated until parturition (Fig. 6a). Maternal blood pressure in CA-Hif-1α pregnant mice decreased following delivery and returned to normal levels one day after birth (Fig. 6a), consistent with and characteristic of the preeclamptic phenotype.

Along with maternal high blood pressure, maternal glomeruloendotheliosis is a classic hallmark of preeclampsia in humans^57^. To determine if trophoblast-specific expression of CA-Hif-1α mice resulted in altered maternal renal morphology and function, maternal kidneys were evaluated. Maternal kidneys from GFP controls at E19.5 displayed normal architecture (Fig. 6b,d). Maternal kidneys from CA-Hif-1α mice at E19.5, however, had discernable collagen deposition in the glomeruli, as well as occlusion of capillary lumens, as indicated by Masson-Trichrome staining (Fig. 6b,c). PAS staining further confirmed these results and also indicated that CA-Hif-1α mothers have thinner glomerular basement membranes (Fig. 6d,e), indicative of glomeruloendotheliosis. To determine if the observed glomerular changes in CA-Hif-1α mice are accompanied by altered kidney function and proteinuria, glomerular barrier function was assessed by measuring urinary proteins. Consistent with the observed glomerular pathological damage, total urinary proteins were significantly increased in CA-Hif-1α mice (Fig. 6f). Additionally, the urinary albumin/creatinine ratio (ACR), indicative of proteinuria, was also are significantly elevated CA-Hif-1α mice compared to GFP control mice (Fig. 6g). Collectively, our data demonstrate that glomerular damage and dysfunction occurs in CA-Hif-1α mice and indicates that the prolonged presence CA-Hif-1α, specifically in placental trophoblasts, promotes kidney damage and results in proteinuria, consistent with a preeclamptic phenotype^57,58^.

While several animal models have been shown to exhibit some of the pathological effects present in human preeclampsia, these models often do not represent the *physiological* aspects that have been identified^57,58^. Reduced uterine perfusion pressure (RUPP), administration of autoimmune antibodies or infusion of pro-inflammatory cytokines to induce a pathological preeclamptic phenotype, are not pregnancy specific and result in systemic inflammation^59,60^. COMT and Elabela gene knockout mouse models have also been shown to display several characteristics of the preeclamptic phenotype; however, both the fetus and placenta have the cognate genes ablated and these models can have fetal defects that are not seen in preeclampsia^61,62^. In addition, maternal administration of adenoviral sFLT1 or adenoviral Hif-1α have been shown to induce preeclampsia like symptoms as well. However, these models are not pregnancy specific, do not induce defects in spiral artery remodeling, and can cause hypertension in the absence of pregnancy^63,64^. Furthermore, maternal administration of adenoviral Hif-1α suggests that Hif-1 overexpression is sufficient for the development of HELLP-like syndrome, a rare form of preeclampsia; however, this was *independent of the presence of the placenta*^64^. In addition, maternal adenoviral Hif-1α infects several organ systems and causes substantial liver damage, which is not due to Hif-1α expression, but rather due to nonspecific viral hepatitis^64^. Interestingly, it was suggested that the liver damage induced by maternal adenoviral Hif-1α administration is the likely source of elevated sFLT1 in this and potentially other maternal adenoviral models that produce pathological symptoms of preeclampsia, and not the placenta^64^. In CA-Hif-1α mice, endothelial dysfunction is present, as the maternal spiral arteries are not remodeled (Fig. 5); however, we did not observe an elevation in sFLT1 in the placenta by *in situ* hybridization or in maternal serum by ELISA, compared to GFP control mice (data not shown). This is consistent with studies that have reported that levels of sFLT1 can be variable or may originate from non-placental sources, and may be due to the trophoblast specificity of our model^65,66^.

In summary, our studies have prolonged the expression of CA-Hif-1α beyond its normal developmental window, as is observed in numerous studies of human preeclampsia, exclusively in trophoblast cells of the placenta. This prolonged expression of Hif-1α, specifically in trophoblasts, resulted in pregnancy-specific placental disorganization, altered placental lineage specification, and inhibition of trophoblast differentiation with endothelial dysfunction and failure to remodel the maternal spiral arteries. These physiological changes in CA-Hif-1α mice, that have also been shown to occur in human preeclampsia, lead to classic preeclamptic pathophysiology of maternal glomeruloendotheliosis, proteinuria, maternal hypertension with post birth resolution and fetal growth restriction^57^. Our findings identify trophoblast Hif-1α as a critical molecular mediator of placental development and indicate that *prolonged expression of Hif-1α*, *explicitly in trophoblast cells of the placenta*, induces maternal and fetal physiological and pathophysiological characteristics that significantly recapitulate that of preeclampsia with FGR and thereby establishes a mouse model that closely resembles the human condition.

## Methods

Detailed materials and methods are described in Supporting Information.

All trophoblast-specific gene transfer experiments involving mice in this study were conducted under a Wright State University IACUC-approved protocol.

### Cloning

The lentiviral construct pLB2V5 was generated by replacing the green fluorescent protein (GFP) gene of pLv-CMV-[GFP]-V5 with the multiple cloning site of pBSSK+^38^. pLB2V5-[CA-Hif-1α] was cloned by ligating the mouse *Hif-1*α triple site-directed mutant cDNA from pc3-Hif-1α3XSDM into the pLB2V5 vector by restriction enzyme digest and confirmed by sequencing (Cleveland Genomics, Cleveland, OH)^34^.

### Western Blotting

Individual placentas from GFP (n = 3 dams) and CA-Hif-1α mice (n = 3 dams) were collected on ice at E19.5 in ice cold 1X RIPA buffer supplemented with protease inhibitor cocktail and proteosome inhibitor MG-132 (Sigma, M7449). Tissues were homogenized on ice for 15 seconds using a Tissue Tearor homogenizer (Biospec). Placental homogenates and whole cell lysates were sonicated for 15 seconds and then centrifuged for 10 min at 4 C to remove debris and used for Western blotting as previously described^19,67–72^.

### Luciferase Assay

To determine Hypoxia Response Element (HRE) reporter activity, COS-7 cells were transiently transfected with 0.2 µg pRL-SV40-promoter constitutive reporter plasmid, 1 µg Pgk1-HRE luciferase reporter plasmid, 1 µg of plasmid (either pLB2V5, pLB2V5-[CA-Hif-1α], or pc3-Hif-1α3XSDM) and 5 µl Metafectene reagent. Luciferase reporter activity was determined using the Dual Luciferase Assay system 24 hours later, according to the manufacturer’s instructions (Promega)^73^.

### Blood Pressure Analysis

Blood pressures were measured and recorded via tail cuff plethysmography using the CODA Non-Invasive Blood Pressure System (Kent Scientific)^55,74,75^.

### Induction of Superovulation, Pseudopregnancy and Embryo Culture

C57BL/6 females were superovulated and mated as previously described^38^. Copulation plugs found the following morning were designated as E0.5. Superovulated C57BL/6 female embryos were collected at E1.5 (2-cell) in M2 media and cultured in pre-equilibrated 20 µl KSOM AA microdrops at 37.5 °C and 5.5% CO2 until E3.5 blastocysts^38^. ICR females were mated with vasectomized ICR males to generate pseudopregnancy, checked for copulation plug the next morning and designated as 0.5 d.p.c.^38^.

### Lentiviral Production, Blastocyst Infection and Embryo Transfer

293FT cells were transfected with pLB2V5-[CA-Hif-1α] or pLv-CMV-[GFP]-V5 and Virapower packaging mix using Metafectene. LB2V5-[CA-Hif-1α] or Lv-CMV-[GFP]-V5 lentivirus was collected, concentrated with PEG-it precipitation solution and titered using HIV-1 p24 Antigen ELISA 2.0, as previously described^38^. Blastocysts were transduced at a final infective concentration of 6,800 ng/ml and 1,200 ng/mL, consistent with previously reported viral titers^35–39,76^. Lentiviral infection of blastocysts and subsequent embryo transfer into pseudopregnant females via NSET were performed as previously described^38^.

### Immunohistochemistry

Immunohistochemistry on paraffin embedded tissue was performed as previously described^44^, using antibodies against V5-epitope (1/100;Millipore, AB3792) and Epcam (1/250;Abcam, ab71916). DAB (Dako, K3468) was used as the chromogenic substrate, and slides were counterstained in Nuclear Fast Red (Vector Labs; H-3403), cleared and mounted using Cytoseal 60 (Thermo-Scientific; 8310-4).

### *In Situ* Hybridization

Digoxigenin-labeled riboprobes were prepared according to the manufacturer’s instructions (Roche, USA). Riboprobes for *Aldh1a3*,*Tpbpa*, *Gcm1*, *Syna*, *Prl3b1*, *Prl2c2*, and *Ascl2* have been previously described^45–49,51,77–80^. Riboprobes for *Pecam1* transcribed from plasmids that were a kind gift from Dr. Jay Cross (University of Calgary, Calgary, Canada). For detection of mouse *Hif1*α, the riboprobe template was amplified by RT-PCR utilizing forward and reverse primers incorporating T3 and T7 RNA polymerase recognition sites, respectively. The primer sequences were, 5′-AATTAACCCTCACTAAAGGGCAGTCGACACAGCCTCGATA (Forward/T3) and 5′-TAATACGACTCACTATAGGGTTTGGAGTTTCCGATGAAGG (Reverse/T7) and amplify an amplicon of 670 bp.

### Placental Histological Staining and Analysis

Alkaline phosphatase (AP) staining to identify maternal blood spaces and isolectin staining to identify fetal blood spaces were conducted as previously described^44^. Periodic Acid Schiff (PAS) staining to identify glycogen accumulation was conducted according to the manufacturer’s protocol (Sigma-Aldrich). Semi-quantitative assessment of placental layers and *in situ* hybridization staining in placenta sections was conducted using NIH ImageJ software. Different layers of the placenta and regions of staining for individual gene markers were outlined and measured manually for layers (in pixels) and using threshold adjustment and selection of threshold area by the software, using Image J, and assessed by two independent observers in a blinded fashion. Area was calculated as a proportion of the total measured area of each of the placentas and reported as % area. At least three independent pictures representing three independent placentas, were measured for each gene or layer quantified.

### Kidney Analysis

Kidneys from dams carrying GFP or CA-Hif-1α infected embryos were isolated at E19.5 and fixed in 4% PFA for embedding in paraffin. To assess kidney pathology, tissue processing, embedding, sectioning and histological staining with hematoxylin and eosin (H&E), periodic acid Schiff (PAS) without diastase and Masson’s trichrome staining were conducted by Reveal Biosciences (San Diego, CA). To assess kidney damage and function, spot urine samples were collected and analyzed from E18.5 and E19.5. Total urinary protein was measured using a bicinchoninic acid (BCA) protein assay (Pierce). Protein concentrations were calculated by extrapolation of values from a protein standard curve. The p-value for total urinary protein was p = 0.0175**. To confirm kidney dysfunction and detect the presence of proteinuria, urinary albumin and creatinine were measured by ELISA (EXOCELL). Microalbuminuria was determined by calculation of the albumin/creatinine ratios (ACR). The p-value for ACR was p = 0.0326**.

### Statistical Analysis

Statistical significance was determined at 0.05. Comparison of fetal and placental weights was performed by mixed model analysis, p = 0.030. Maternal blood pressure analysis was performed by Sidak’s Multiple Comparison test using Prism software. The p-values for blood pressure were E17.5, *p = 0.0274; E18.5, *p = 0.0005; E19.5, *p = 0.0006 and are identified with asterisks. For the luciferase assay, a one way ANOVA and Tukey’s multiple comparison test was used. The p-values were pLB2V5control vs pc3-Hif-1α3XSDM, *p < 0.0001; pLB2V5control vs pLB2V5-CA-Hif-1α, *p < 0.0001.

## Supplementary information

Supporting information
