# Supplementary material for: Trophoblast-Specific Expression of Hif-1α Results in Preeclampsia-Like Symptoms and Fetal Growth Restriction
Source: Sci Rep. 2019 Feb 26;9:2742. doi: 10.1038/s41598-019-39426-5 (PMC6391498; doi:10.1038/s41598-019-39426-5)

**SUPPORTING INFORMATION TITLE PAGE**

Title: Trophoblast-Specific Expression of Hif-1α Results in Preeclampsia-Like Symptoms and Fetal Growth Restriction.

Author List

Renee E. Albers

Melissa R. Kaufman

Bryony V. Natale

Chanel Keoni

Kashmira Kulkarni-Datar

Sarah Min

Clintoria R. Williams

David R.C. Natale

Thomas L. Brown

**Supporting Information**

**METHODS**

**Vertebrate Animals**

All animal procedures were performed in accordance with and approval of the Wright State University Institutional Animal Care and Use Committee (IACUC).

# Materials

The plasmid construct pc3-Hif-1α3XSDM, was generously provided by Dr. Christina Warnecke of Universität Erlangen-Nürnberg, Germany (34). The 293FT cell line, Virapower packaging mix (K495000) and Alexa Fluor 594 secondary antibody (A-11072) for immunocytochemistry were purchased from Invitrogen. Metafectene transfection reagent was obtained from Biontex (T020-1.0). The 5X PEG-it virus precipitation solution was acquired from System Biosciences LLC (LV810A-1). HIV-1 type 1 p24 Antigen 2.0 ELISA kit, used for viral titering, was obtained from ZeptoMetrix Corporation (0801002). C57BL/6 male and female mice, ICR female mice, and vasectomized ICR male mice were purchased from Taconic Biosciences (Germantown, NY) and Charles River Laboratories (Raleigh, NC). Pregnant Mare’s Serum gonadotropin (PMSg) (G4877), Human chorionic gonadotropin (hCG) (C1063), mineral oil (M8410), and Hoechst dye (62249) were acquired from Sigma Aldrich. Pregnant Mare’s Serum gonadotropin (PMSg) (493-10) was also purchased through Lee Biosolutions. KSOM AA with phenol red embryo culture media (GSM-5140) was obtained from MTI-Global Stem. Anti-V5 epitope polyclonal antibody (AB3792), polybrene (TR-1003-G), EmbryoMax Acidic Tyrode’s solution (MR-004-D) and EmbryoMax M2 Media (MR-015-D) were obtained from Millipore Sigma. The Non-Surgical Embryo Transfer Device (NSET) was purchased from ParaTechs Corporation (60010). Optimum cutting temperature (OCT) media was obtained from Tissue-Tek (4583). Primary monoclonal anti-Hif-1α antibody (NB100-105) and primary polyclonal anti-Hif-2α antibody (NB100-122S) were obtained from Novus Biologicals. Primary mouse monoclonal Pdk1 antibody (4A11F5) was acquired from Santa Cruz Biotechnology. Anti-pan actin mouse monoclonal antibody (LMAB-C4) was obtained from Seven Hills Bioreagents. Secondary antibodies (anti-mouse and anti-rabbit) were purchased from Promega (W402B and W401B, respectively). Vectashield Antifade mounting medium with DAPI (H-1200) was obtained from Vector laboratories.

**Cloning and Cell Culture**

The lentiviral construct pLB2V5 was generated by replacing the green fluorescent protein (GFP) gene of pLv-CMV-[GFP]-V5 with the multiple cloning site of pBSSK+ (38). pLB2V5-[CA-Hif-1α] was cloned by ligating the mouse *Hif-1*α triple site-directed mutant cDNA from pc3-Hif-1α3XSDM into the pLB2V5 vector by restriction enzyme digest using BamHI and ApaI and confirmed by DNA sequencing (Cleveland Genomics, Cleveland, OH). 293FT cells were cultured in HyClone DMEM/High glucose (GE Healthcare Life Sciences, SH30022.01), 10% heat-inactivated fetal bovine serum (Biowest, S01520), 1% antibiotic-antimycotic (Thermo Scientific, SV30079.01), 1mM sodium pyruvate (Sigma, S8636), 2mM glutaGRO Supplement (Mediatech, Inc., 25-015-CI), 0.1mM NEAA Mixture (Lonza, 13-114E) and 500ug/mL G418 (InvivoGen, ant-gn) (38). COS-7 cells were cultured in DMEM/High glucose, 10% heat inactivated fetal bovine serum, and 1% antibiotic-antimycotic. HEK293 cells were cultured as previously described (67). All cell types were passaged at 80-90% confluence.

**Western Blotting**

Placentas from GFP and CA-Hif-1α mice were collected on ice at E19.5 in ice cold 1X RIPA buffer supplemented with protease inhibitor cocktail and proteosome inhibitor MG-132 (Sigma, M7449). Tissues were homogenized on ice for 15 seconds using a Tissue Tearor homogenizer (Biospec). COS-7 whole cell lysates, treated with vehicle or 300 ng/ml CoCl_2_ were collected after 24hrs in 1X ice cold RIPA buffer supplemented with protease inhibitor cocktail and proteosome inhibitor MG-132 (Sigma, M7449). Placental homogenates and whole cell lysates were sonicated for 15 seconds and then centrifuged for 10 min at 4C to remove debris. The Bradford method was used to determine protein concentration (68,69). Placental and whole cell lysates were incubated at 95C for 10 minutes in 1X Laemmli sample buffer (70-72). Proteins (150 µg) were electrophoresed on a 10% SDS-polyacrylamide gel and subsequently transferred to an Immobilon-P Transfer Membrane (19). Ponceau S staining was used to confirm protein transfer. Following transfer, the membrane was blocked for 1 hour in blocking buffer (1X PBS pH 7.4 containing 0.05% Tween-20 and 5% fat-free dry milk) at room temperature. Subsequently, the blot was incubated with primary Hif-1α, Hif-2α or Pdk1 antibody (1:1,000) at 4C overnight with rocking or anti-pan actin clone C4 antibody (1:10,000) at room temperature for 1hr. Antibodies were diluted with 1xPBS with 0.05% Tween-20 and 5% fat-free dry milk at 4C overnight. The membrane was washed with 1X PBS containing 0.05% Tween 20, incubated with secondary antibody (1:25,000) diluted with 1xPBS with 0.05% Tween 20 and 5% fat-free dry milk for 1 hour and then washed with 0.05% Tween 20 in 1X PBS (19,67-72). Membranes were developed using the Super-Signal West Pico Chemiluminescent Substrate kit (Thermo Scientific, 34580) and exposed to x-ray film.

**Blood Pressure Analysis**

Blood pressures were measured and recorded via tail cuff plethysmography using the CODA Non-Invasive Blood Pressure System (Kent Scientific) (74,75). Adult female ICR mice were acclimated to the tail cuff at least three separate days, for 10 minutes, prior to inducing pseudopregnancy. Baseline blood pressures were recorded at 0.5 d.p.c. (days post coitum) on plug-positive ICR mice. Tails of pseudopregnant ICR mice were allowed to acclimate to 32C for 10 minutes and then given 2 initial acclimation cycles prior to collecting acceptable readings. Those identified within normal blood pressure range were used for subsequent non-surgical embryo transfer (NSET). Following embryo transfer, pregnant ICR mice were acclimated to tail cuff for 10 minutes, every other day until E12.5 (embryonic day), after which, readings were recorded on E14.5, E15.5, E17.5, E18.5, E19.5 and 1day post birth.

**Fetal, Placental and Kidney Collection**

Fetuses and placentas were dissected at E14.5, E19.5 or at time of birth in ice cold 1X PBS and wet weights were measured. After weighing, placentas were cut in half and ½ fixed in 4% paraformaldehyde/1X PBS at 4^0^C overnight and the other ½ half was placed in RNAlater and stored at 4C until analyzed. Following fixation, tissues were washed several times in ice cold 1X PBS and then dehydrated through 50% and 70% ethanol prior to xylene and paraffin embedding (38,45,46,53,77,78). Kidneys were dissected at E19.5, bisected and fixed as described above.

**In Situ Hybridization**

Placentas for in situ hybridization were dissected at E14.5 or birth and fixed overnight, embedded in paraffin and sectioned (7µm) as previously described (80). For *in situ* hybridization, sections underwent deparaffinization in xylene followed by rehydration through a graded ethanol series ending in 1X PBS. Sections were then post-fixed in 4% paraformaldehyde/1X PBS, treated with proteinase K (15 µg/ml for 20 minutes at room temperature), acetylated for 10 minutes (acetic anhydride, 0.25%; Sigma, 320102) and hybridized with DIG-labeled probes overnight at 65C. Hybridization buffer contained 1X salts (200mM NaCl, 13 mM Tris, 5 mM sodium phosphate monobasic, 5 mM sodium phosphate dibasic, 5 mM EDTA), 50% formamide, 10% (w/v) dextran sulfate, 1 mg/ml yeast tRNA (Sigma), 1X Denhardt’s [1% (w/v) bovine serum albumin, 1% (w/v) Ficoll, 1% (w/v) polyvinylpyrolidine)] and cRNA probe (final dilution of 1:2,000 from a reaction with 1 µg template DNA). Post-hybridization washes were followed by an RNase treatment [400mM NaCl, 10mM Tris (pH 7.5), 5 mM EDTA, 20 µg/ml RNase A] for 30 minutes at 37C. After blocking, sections were incubated overnight at 4C in alkaline phosphatase-conjugated, anti-DIG antibody diluted 1:2,500 in blocking solution. Sections were washed and positive staining was detected as a purple-blue precipitate using alkaline phosphatase in coordination with NBT/BCIP (45). Sections were then counterstained with Nuclear Fast Red, dehydrated and mounted in Cytoseal 60 (VWR) permanent mounting media.

**Supplementary Figure Legends**

**Figure S1. Lentiviral V5 epitope is expressed in GFP and CA-Hif-1α-transduced placentas.** V5 epitope staining by immunohistochemistry was conducted to determine localization of the lentiviral constructs in GFP and CA-Hif-1α placentas at E14.5. Positive staining was observed in the labyrinth as well as in regions of the decidua that are consistent with invading/migrating trophoblast cells. Scale bar = 500 μm.

**Figure S2. Western Blot- raw data –anti Hif -1 and anti pan actin in COS-7 cells.**

COS-7 cells in ambient oxygen were treated with mock (1), CoCl_2_(2), or infected with pLv-CMV-GFP (3), or different viral stocks of pLB2V5-CA-Hif-1α (4-10). Whole cell lysates (100μg) were analyzed by Western blot for Hif-1α (top) and stripped and reprobed with pan-actin antibody as a loading control. (see **Fig.1a** main text)

**Figure S3. Western Blot- raw data-GFP and CA-Hif-1α** **placental lysates with anti-Hif-1α.**

(see **Fig. 1e** main text)

**Figure S4. Western Blot- raw data-GFP and CA-Hif-1α placental lysates with anti-PDK1.**

(see **Fig. 1e** main text)

**Figure S5. Western Blot- raw data-GFP and CA-Hif-1α** **placental lysates with anti-Hif-2α.**

(see **Fig. 1e** main text)

**Figure S6. Western Blot- raw data-GFP and CA-Hif-1α** **placental lysates with anti-pan actin.**

(see **Fig. 1e** main text)

**Figure S7. Litter size and Fetal/placental ratio.**

Litter size (GFP dams n=8; pups n= 23 and CA-Hif-1α dams n=6; pups n=21) was analyzed by unpaired Students t-test **(a)**. For measurements of fetal/placental weight ratio, matching fetal and placental weights for 8-10 pups per stage for CA-Hif-1α and 20-22 pups per stage for GFP were used **(b)**. Two-way ANOVA was used to determine statistical difference between the groups and indicated no statistical differences.

**Figure S8. Distribution of relative areas in GFP and CA-Hif-1α placentas.** Shows the distribution of the three layers of the mouse placenta in GFP and CA-Hif-1α placentas at E14.5 and at birth as determined by measuring the area of each layer, relative to the entire area of the placenta. Values are presented as a percentage and represent measurements of 3-5 sections from n= 3-5 individual placentas from each group and at each stage.

**Figure S9. Placental morphology and lineage marker analysis. (a)** Representation of the mouse placenta showing labyrinth, junctional zone and decidual layers. In addition, the trophoblast subtypes found in each layer and gene markers used to identify them are indicated. **(b)** Table showing placental cell type, marker and histological or gene expression analysis used to identify them in this study.

**Figure S10. Branching of fetal vessels in the chorionic plate is limited in CA-Hif-1α placentas.** Immunohistochemistry for the labyrinth progenitor cell marker, Epcam showed staining that was restricted primarily to the chorionic plate of the labyrinth layer in both GFP **(a,c)** and CA-Hif-1α **(b,d)** placentas at both E14.5 **(a,b)** and birth **(c,d)**. Binding of Isolectin-BS1 was used to identify fetal blood vessels in the labyrinth layer of GFP **(e,g)** and CA-Hif-1α **(f,h)** placentas at E14.5 **(e,f)** and birth **(g,h)**. Arrowheads indicate fetal branch points **(a-h).** Scale bar = 500 μm.

**Figure S11. Ascl2 progenitors are unchanged and PAS staining highlights differences in glycogen trophoblasts.** *Ascl2* expression at E14.5, shown by *in situ* hybridization, identifies progenitors of the junctional zone and was similar between GFP **(a)** and CA-Hif-1α **(b)** placentas. PAS staining of GFP **(c,e)** and CA-Hif-1α **(d,f)** placentas at E14.5 highlighted glycogen trophoblasts in the junctional zone. Boxed regions in **(c,d)** are shown at higher magnification in **(e,f).** Arrowheads show glycogen trophoblasts with a foamy appearance and clear cytoplasm **(e,f)**. Scale bar = 500 μm **(a,b,c,d);** Scale bar = 100 μm **(e,f)**.

**Figure S12. Glycogen trophoblasts are more densely packed in CA-Hif-1α placentas.** *Prl3b1* expression is shown by *in situ* hybridization and identifies parietal trophoblast giant cells and spongiotrophoblasts in the junctional zone, as well as sinusoidal trophoblast giant cells in the labyrinth at E14.5. High magnification images of boxed regions in **(a,b)** are shown in **(c,d)** and highlight the morphology of unstained glycogen trophoblast (arrowheads). Scale bar = 500 um **(a,b);** Scale bar = 100 um **(c,d)**.

**Figure S13. CA-Hif-1α placentas exhibit reduced expression of *Prl2c2* *(Plf)*.** *Prl2c2* expression is shown by *in situ* hybridization in GFP **(a,c)** and CA-Hif-1α **(b,d)** at E14.5 **(a,b)** and at birth **(c,d).** CA-Hif-1α placentas **(b)** at E14.5 and birth exhibit a dramatic reduction in Prl2c2 expression compared to control GFP placentas **(a)**. Scale bar = 500 μm.


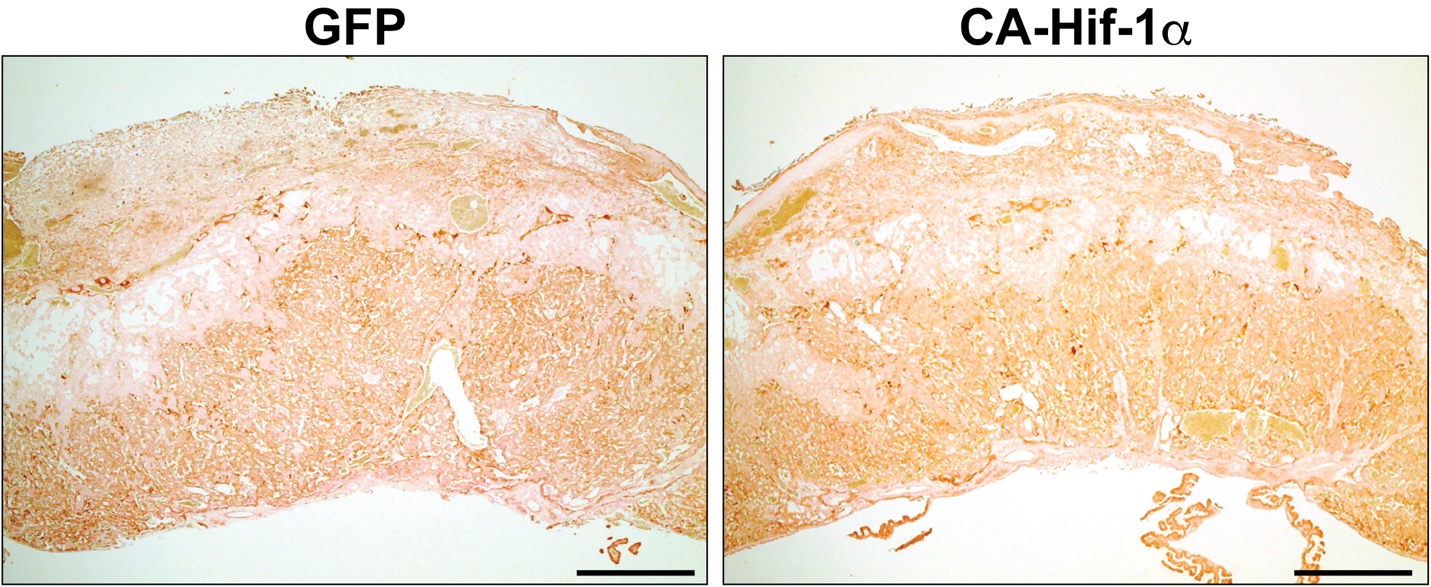


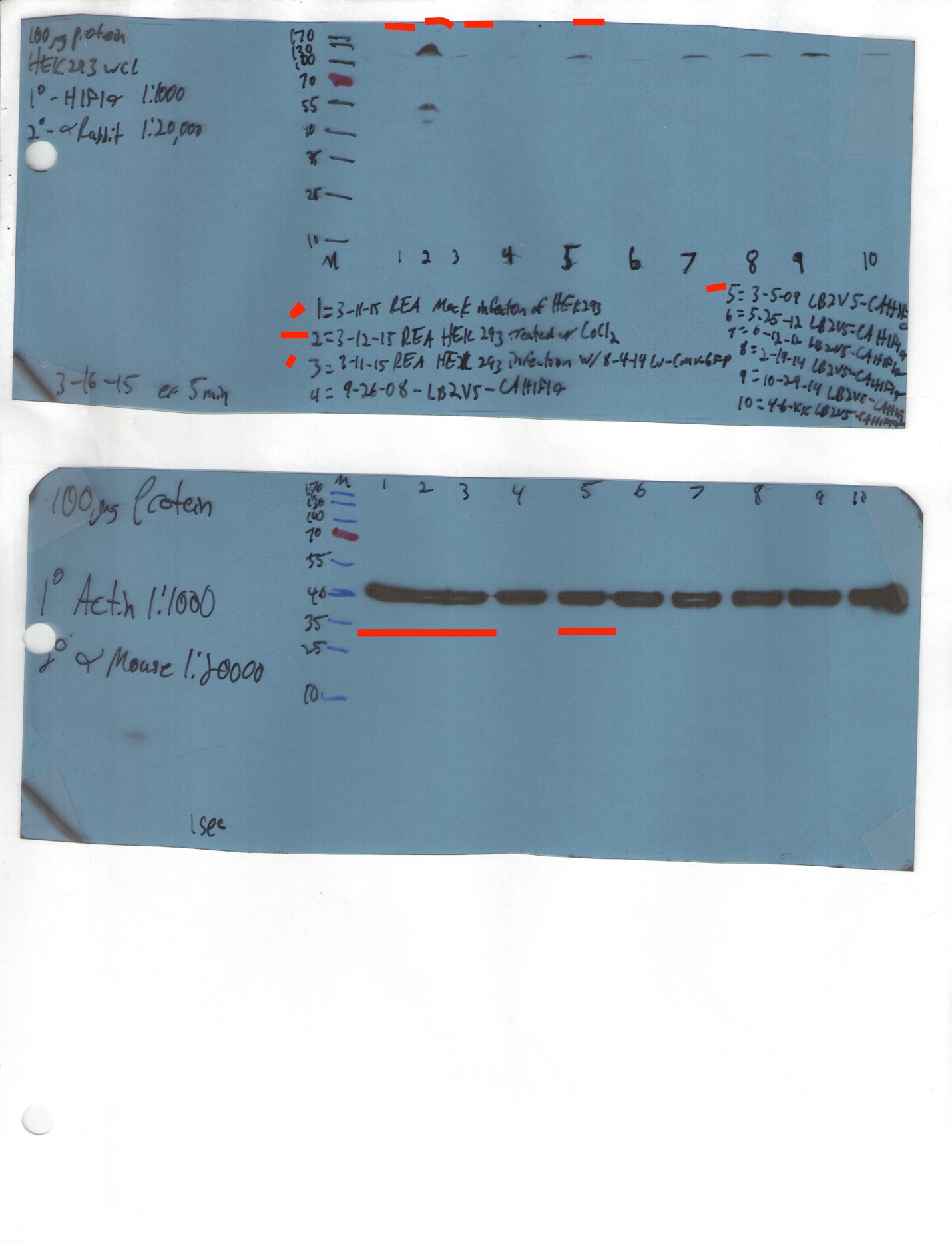


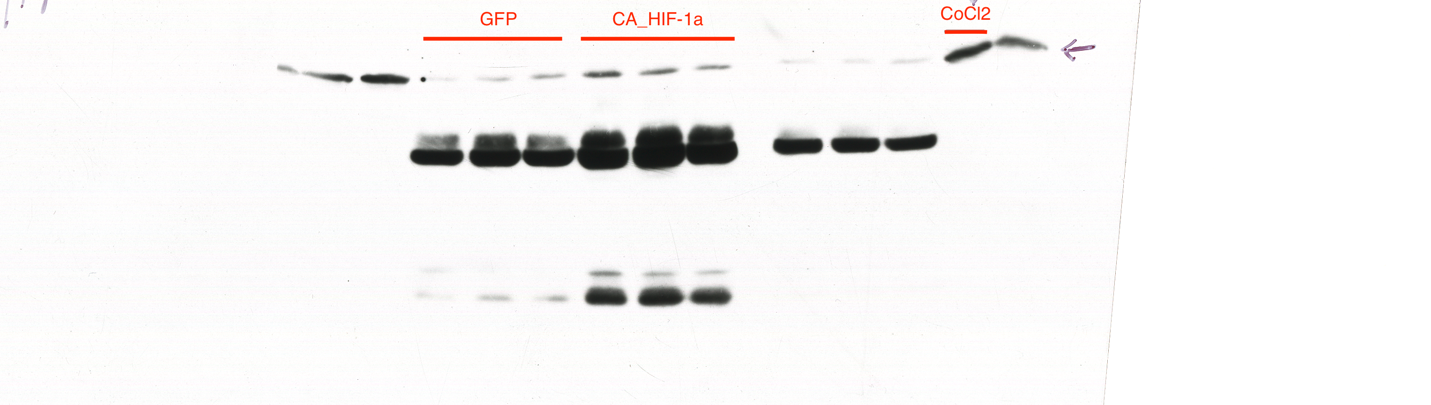


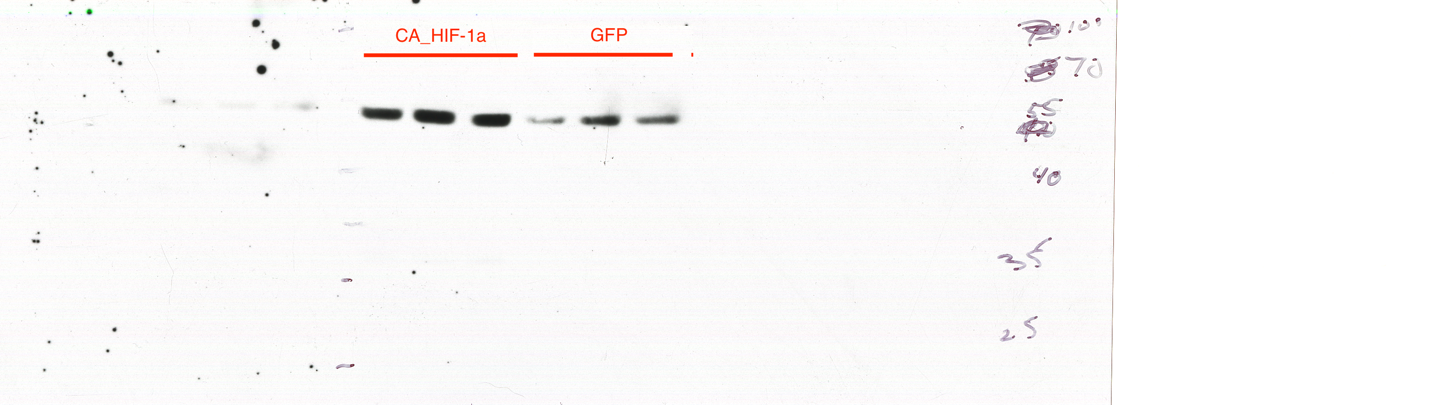


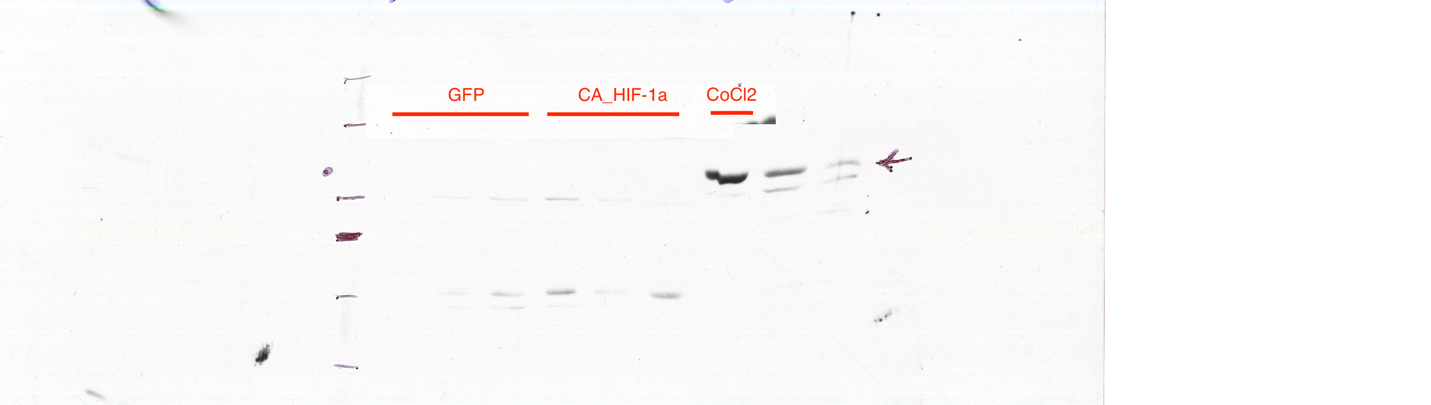


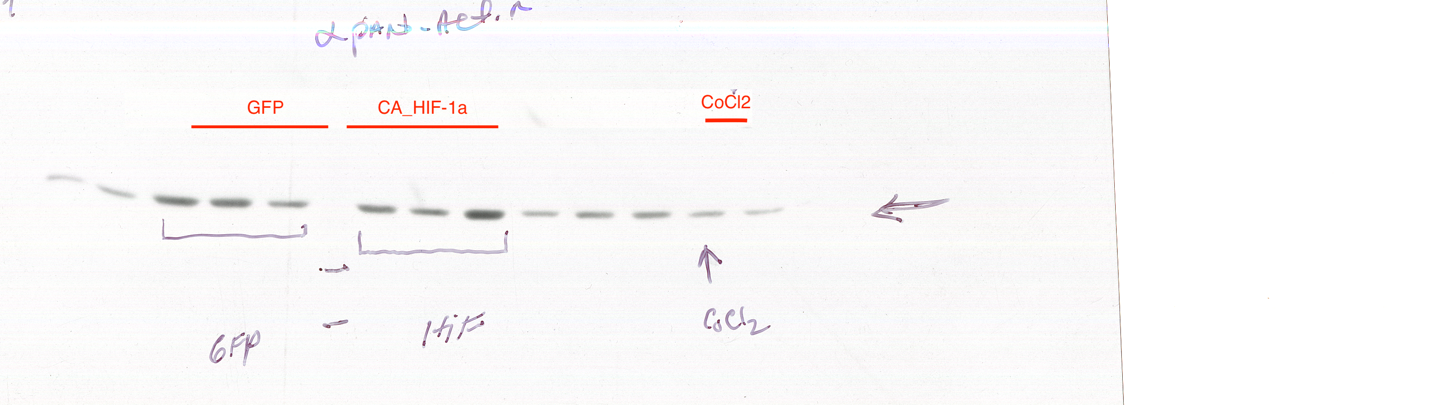

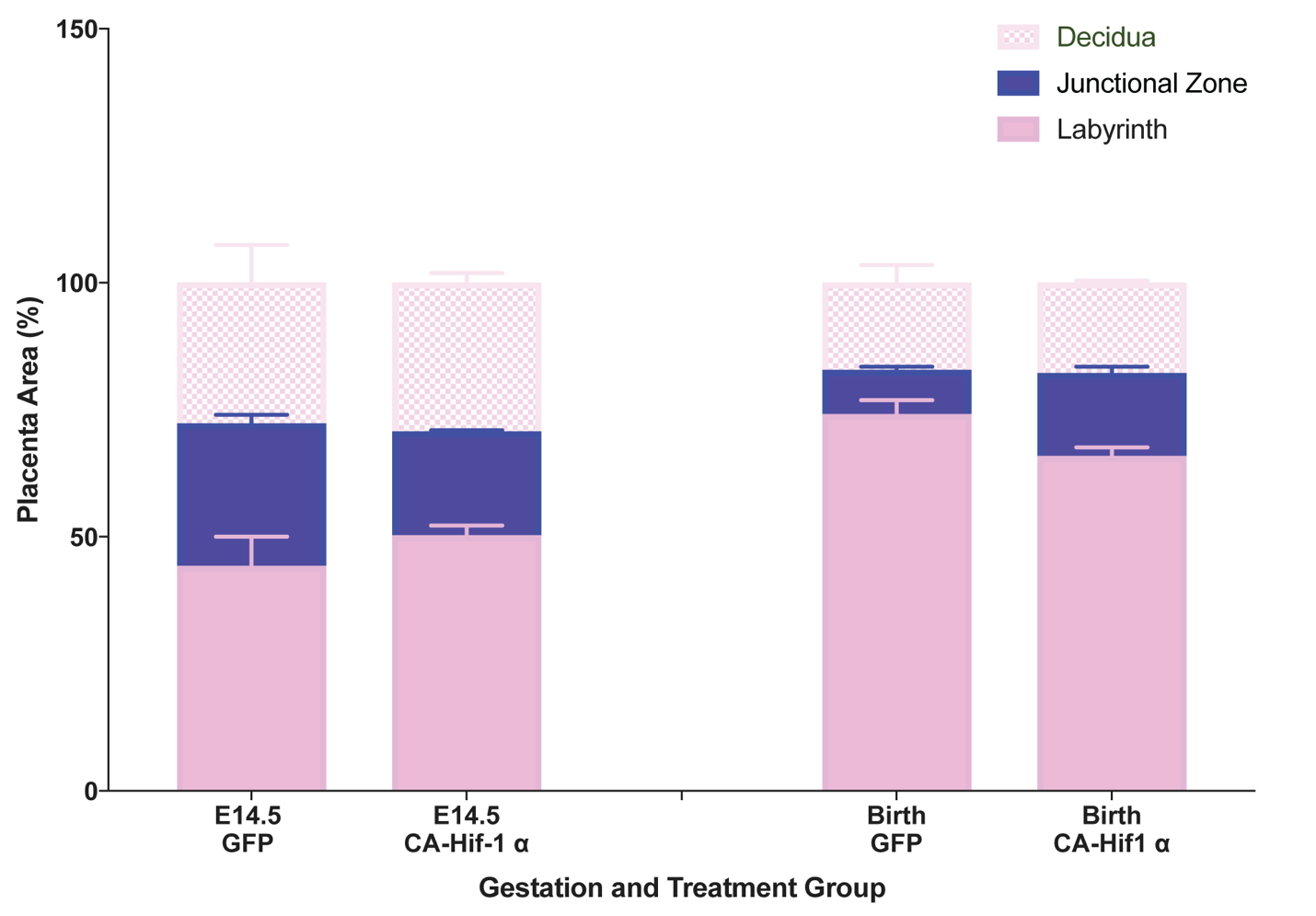


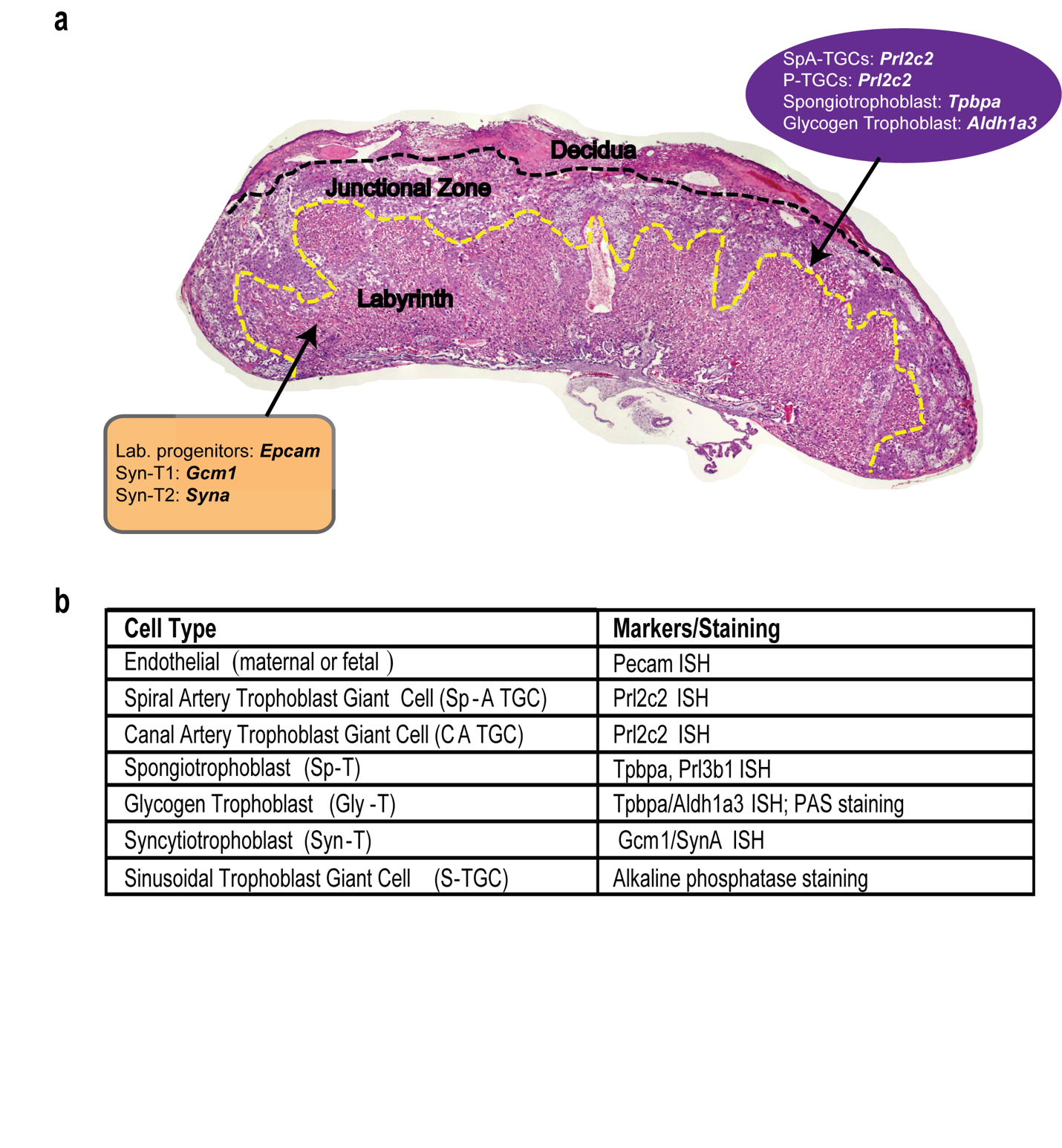


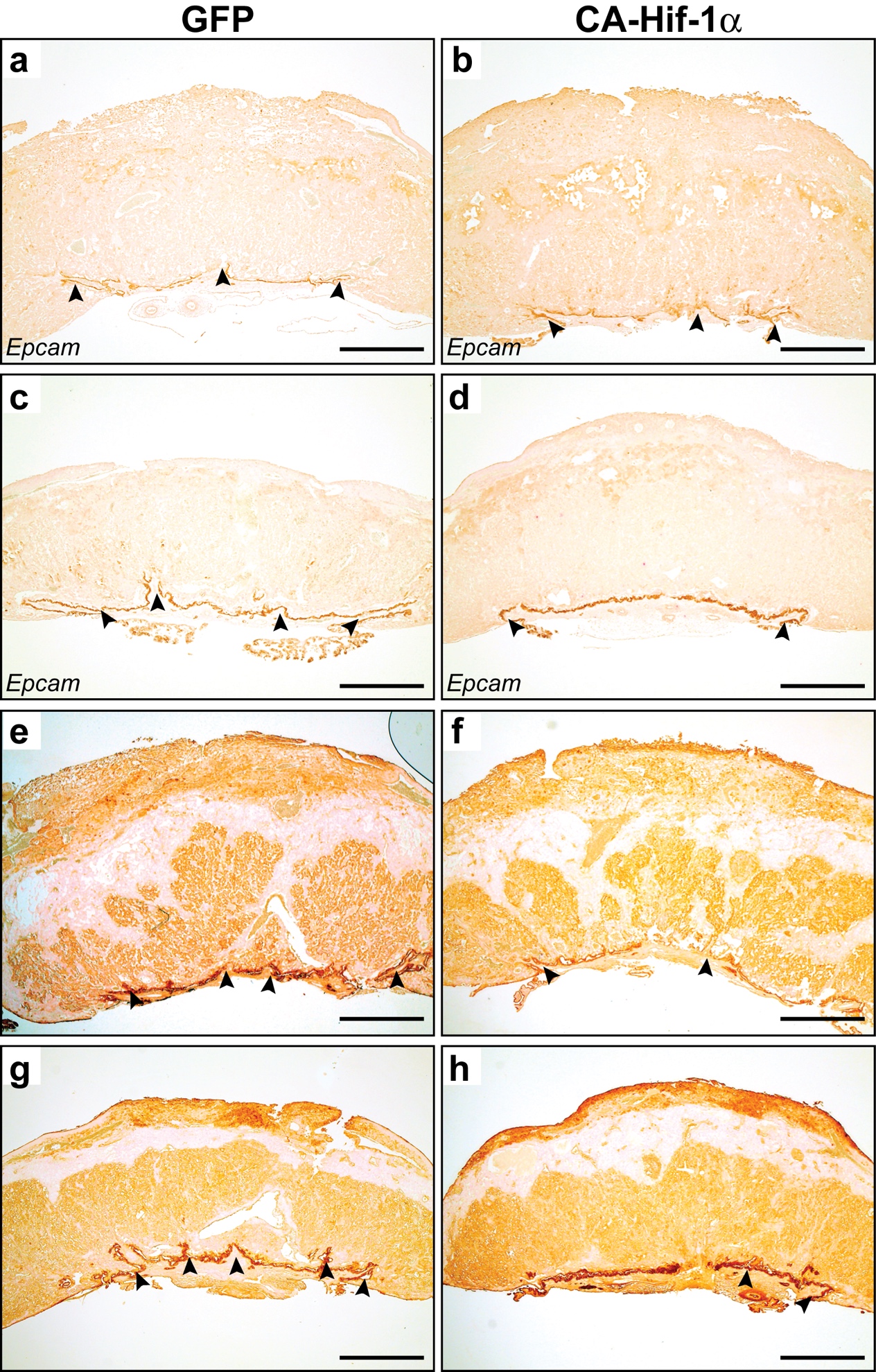


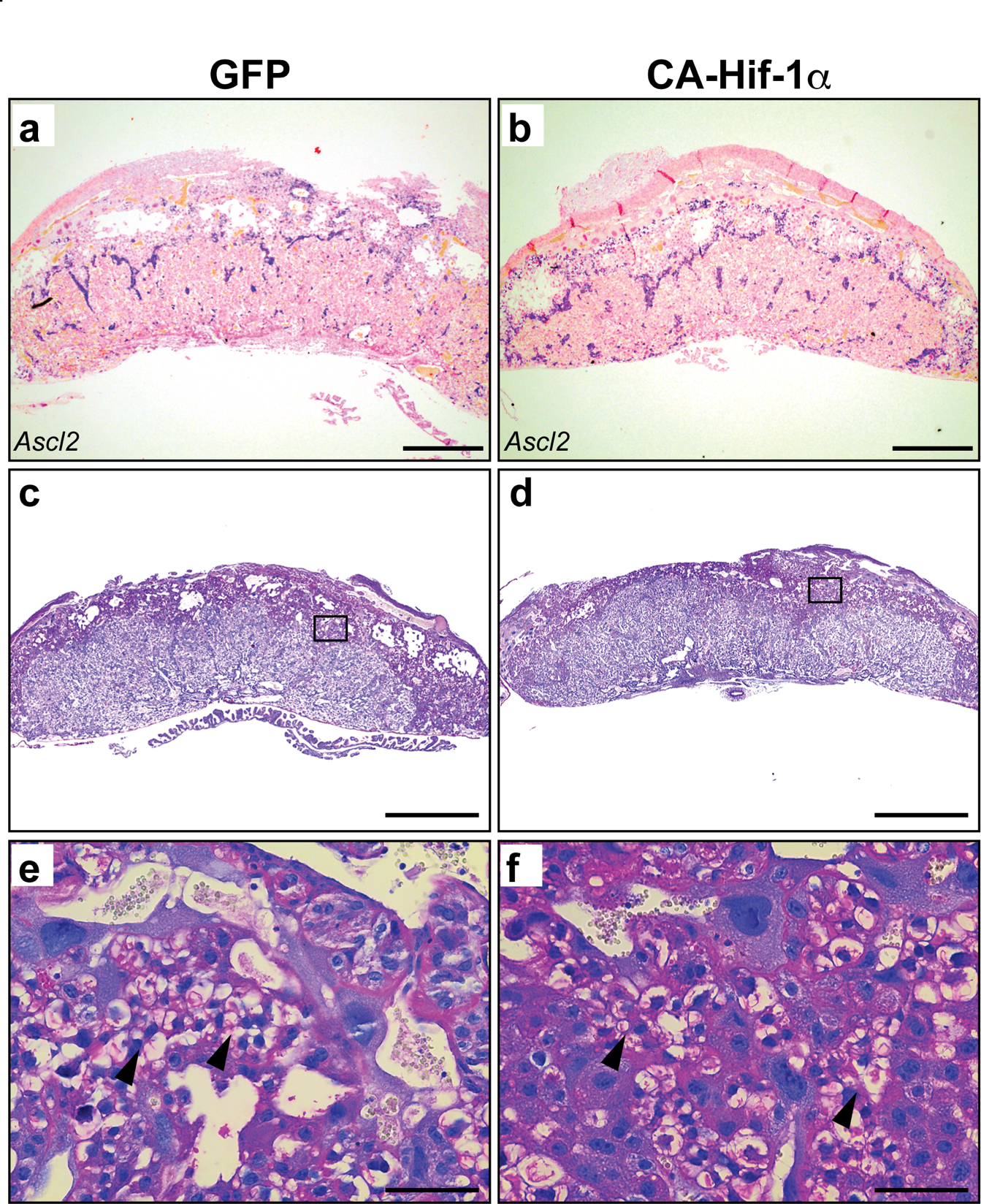


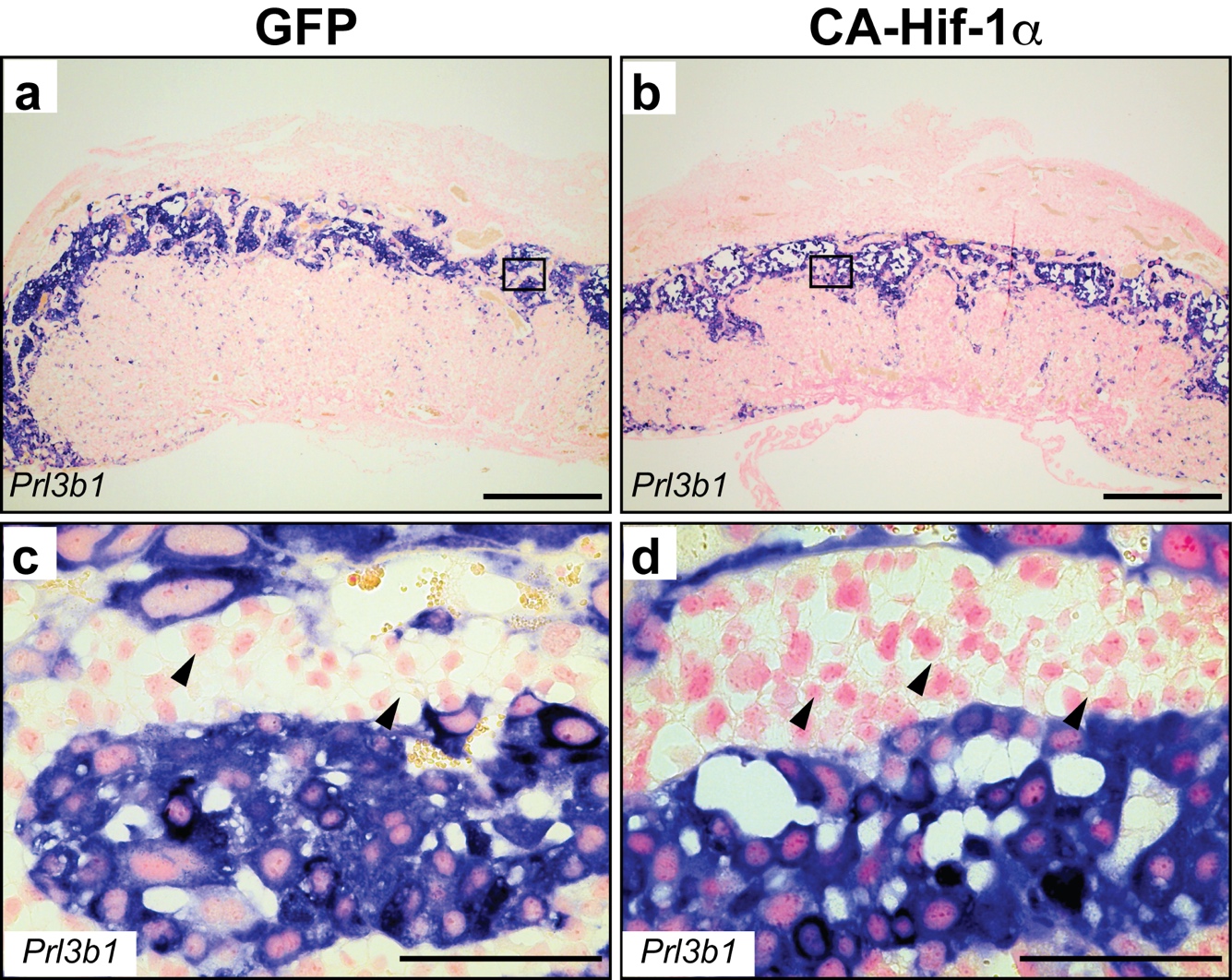


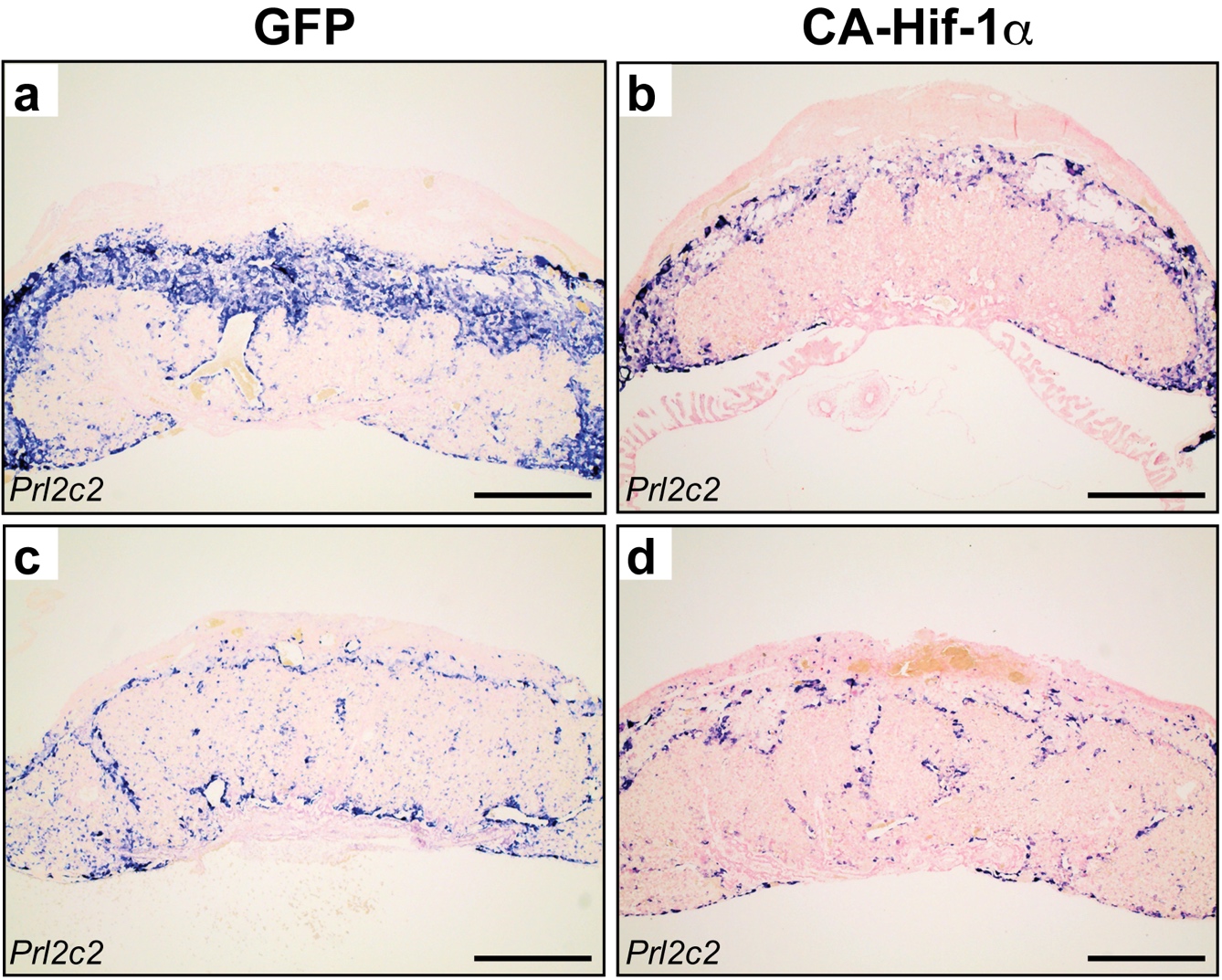

Supplement: Supplementary file 1 — Supporting information [file 41598_2019_39426_MOESM1_ESM.docx]
